# CTX-M β-Lactamase–producing *Klebsiella pneumoniae* in Suburban New York City, New York, USA

**DOI:** 10.3201/eid1911.121470

**Published:** 2013-11

**Authors:** Guiqing Wang, Tiangui Huang, Pavan Kumar Makam Surendraiah, Kemeng Wang, Rashida Komal, Jian Zhuge, Chian-Ru Chern, Alexander A. Kryszuk, Cassidy King, Gary P. Wormser

**Affiliations:** New York Medical College, Valhalla, New York, USA

**Keywords:** Klebsiella pneumoniae, Escherichia coli, extended-spectrum beta-lactamases, beta-lactamases CTX-M-15, pulsed-field gel electrophoresis, multilocus sequence typing, bacteria, antimicrobial resistance

## Abstract

CTX-M extended-spectrum β-lactamase (ESBL)–producing *Klebsiella pneumoniae* isolates are infrequently reported in the United States. In this study, we analyzed nonduplicate ESBL-producing *K. pneumoniae* and *Escherichia coli* clinical isolates collected during 2005–2012 at a tertiary care medical center in suburban New York City, USA, for the presence of *bla*_CTX-M_, *bla*_SHV_, *bla*_TEM_, and *bla*_KPC_ genes. Despite a high prevalence of *bla*_CTX-M_ genes in ESBL-producing *E. coli* since 2005, *bla*_CTX-M_ genes were not detected in *K. pneumoniae* until 2009. The prevalence of CTX-M–producing *K. pneumoniae* increased significantly over time from 1.7% during 2005–2009 to 26.4% during 2010–2012 (p<0.0001). CTX-M-15 was the dominant CTX-M genotype. Pulsed-field gel electrophoresis and multilocus sequence typing revealed high genetic heterogeneities in CTX-M–producing *K. pneumoniae* isolates. This study demonstrates the recent emergence and polyclonal spread of multidrug resistant CTX-M–producing *K. pneumoniae* isolates among patients in a hospital setting in the United States.

CTX-M enzymes are a group of class A extended-spectrum β-lactamases (ESBLs) that are rapidly spreading among *Enterobacteriaceae* worldwide (*1*). Since the initial isolation of CTX-M-1 from a European patient in the late 1980s (*2*), >130 CTX-M allelic variants have been described (http://www.lahey.org/Studies/other.asp#table1). These CTX-M variants have been divided into 5 major phylogenetic groups, CTX-M-1, CTX-M-2, CTX-M-8, CTX-M-9, or CTX-M-25 on the basis of their amino acid sequences (*1**,**2*).

During the past decade, CTX-M enzymes have become the most prevalent ESBL enzymes in clinical *Enterobacteriaceae* isolates, especially in ESBL-producing *Escherichia coli* in Europe, Asia, and South America (*1**,**3*). By contrast, SHV- and TEM-type ESBL enzymes are primarily found in ESBL-producing *K. pneumoniae* and *E. coli* clinical isolates in North America (*3*). In the United States, CTX-M–like ESBL-producing *Enterobacteriaceae* was first reported in 2003, when CTX-M enzymes were detected in 9 *E. coli* clinical isolates from 5 US states (*4*). The spread of CTX-M type ESBL in *Enterobacteriaceae*, however, was not appreciated until 2007 when a Texas study showed a high prevalence of CTX-M ESBL in *E. coli* clinical isolates recovered during 2000–2005 (*5*). Since then, CTX-M–producing *E. coli* isolates have been documented in dispersed US geographic regions (*3**,**6**,**7*). CTX-M enzymes are now the predominant ESBL type in *E. coli* clinical isolates in Texas (*5*), Pennsylvania (*6*), Illinois (*8*), and New York (*9**,**10*).

CTX-M–type ESBL enzymes have also been reported in the United States in some non–*E. coli Enterobacteriaceae* species, such as *Klebsiella* spp. (*5**,**11**,**12*), *Proteus mirabilis* (*5**,**11*), *Enterobacter* spp. (*5*), *Salmonella* spp. (*13*), *Shigella* spp. (*14*), and *Morganella morganii* (*5*). Nevertheless, CTX-M–type ESBL have been principally detected and reported in *E. coli* clinical isolates. To date, <50 CTX-M–producing *K. pneumoniae* isolates have been described in the United States, and the epidemiologic and microbiological data provided have been limited (*5**,**11**,**12**,**15**–**18*). The implications of CTX-M–producing *K. pneumoniae* for laboratory detection, patient care, and public health in the United States remain to be elucidated.

In this study, we investigated the prevalence of SHV-, TEM-, and CTX-M–encoding genes in a large collection of ESBL-producing *K. pneumoniae* and *E. coli* clinical isolates from a tertiary care medical center in suburban New York City in Westchester County, New York, over an 8-year period (2005–2012). Microbiological characteristics of CTX-M ESBL-producing *K. pneumoniae* isolates were examined, and certain clinical/epidemiologic features of patients with these isolates were analyzed. 

## Materials and Methods

### Bacterial Isolates and Phenotypic Detection of ESBLs

Nonduplicate *K. pneumoniae* clinical isolates were recovered from patient specimens during January 2005–July 2012 at the clinical microbiology laboratory of Westchester Medical Center. These included 208 *bla*_KPC_-negative non-*K. pneumoniae* carbapenemase (non-KPC) ESBL-producing or third-generation cephalosporin-resistant *K. pneumoniae* isolates and 228 KPC (*bla*_KPC_-positive)–producing *K. pneumoniae* isolates. In addition, 163 nonduplicate ESBL-producing *E. coli* clinical isolates from the same period were also analyzed for comparison. Isolates were randomly selected to span the entire study year with an approximately equal number of isolates per quarter; only 1 isolate from each patient was chosen and tested. The center is a 643-bed academic tertiary-care medical center in Westchester County, New York. The Institutional Review Board of New York Medical College approved this study.

The bacterial isolates were identified and evaluated for antimicrobial drug susceptibility with the MicroScan WalkAway 96 system (Siemens, Sacramento, CA, USA). ESBL production was phenotypically confirmed by a double-disk or broth microdilution method for suspected ESBL isolates according to the Clinical and Laboratory Standards Institute (CLSI) guidelines (*19*). The antimicrobial drug susceptibility of CTX-M–producing *K. pneumoniae* isolates against selected antimicrobial drugs was also assessed with standardized CLSI disk diffusion and Etest methods. Bacterial isolates were refrigerated on nutrient agar slants or were frozen (−80°C) in MicroBank cryovials containing 20% glycerol (Pro-Lab Diagnostics, Round Rock, TX, USA). For antimicrobial drug susceptibility testing of frozen isolates, fresh subcultures were used per CLSI guidelines.

### PCR Detection of *bla*_CTX-M_, *bla*_SHV_, *bla*_TEM_, and *bla*_KPC_ Genes

For PCR, bacterial genomic DNA was extracted directly from colonies on nutrient slants or from fresh subcultures grown on Trypticase soy agar with 5% sheep blood (TSA II, BBL, Sparks, MD, USA) by boiling a dense suspension of an approximately no. 1 McFarland standard in sterile distilled water. As the DNA template in the PCR assays, 2–3 μL of the boiled cell suspension was used. PCR amplification of *bla*_CTX-M_, *bla*_SHV_, *bla*_TEM_, and *bla*_KPC_ genes in *K. pneumoniae* and *E. coli* clinical isolates was performed by using a consensus primer pair specific to each type of β-lactamase as described (*20**–**22*). A multiplex PCR was developed and used for simultaneous detection of *bla*_CTX-M_ (551 bp) and *bla*_TEM_ (972 bp) genes. Two PCRs were performed for *bla*_SHV_-ESBL and *bla*_KPC,_ respectively. PCRs were carried out by using the HotStart DNA polymerase master mix (QIAGEN, Germantown, MD, USA) with 30–35 cycles at an annealing temperature of 50°C for *bla*_CTX-M_ and *bla*_TEM,_ and 52°C for *bla*_SHV_ and *bla*_KPC._ PCR products were analyzed by agarose gel electrophoresis or by using the QIAxcel system (QIAGEN). The specificity of PCR amplicons on representative isolates was confirmed by DNA sequencing.

### DNA Sequencing

For DNA sequencing, PCR products were purified by using the PCR Purification kit (QIAGEN) or the ExoSAP-IT PCR Clean-up kit (Affymetrix, Cleveland, OH, USA), according to the manufacturer’s instructions. The purified DNA amplicons were sequenced by using an ABI Prism BigDye Terminator (version 1.1) cycle sequencing ready reaction kit on the ABI Prism 3730xl or ABI 3500xl DNA Analyzers (Applied Biosystems, Foster City, CA, USA) in-house, or by a commercial facility (GeneWiz, South Plainfield, NJ, USA). The CTX-M, TEM, and SHV gene sequences were compared with sequences in GenBank by using the NCBI basic local alignment search tool (www.ncbi.nlm.nih.gov/BLAST).

### Multilocus Sequence Typing

Multilocus sequence typing (MLST) was performed by using primers and conditions as described by Diancourt et al. (*23*). PCR products from MLST were sequenced as described above. Allelic profiling and sequence types (STs) were determined by querying the *K. pneumoniae* MLST database maintained by the Pasteur Institute (www.pasteur.fr/recherche/genopole/PF8/mlst/Kpneumoniae.html).

### Pulsed-field Gel Electrophoresis (PFGE)

Pulsed-field gel electrophoresis (PFGE) on CTX-M ESBL–producing *K. pneumoniae* isolates representing each CTX-M genotype was performed as described (*24*). The GelCompare II software, (version 2.0; Applied Maths, Austin, TX, USA) was used to calculate the Dice similarity coefficients and generate dendrograms by cluster analysis with the unweighted-pair group method using average linkages. Pulsotype designations were assigned at the ≥80% profile similarity level.

## Results

### CTX-M in ESBL-producing, non-KPC *K. pneumoniae* Clinical Isolates

Of the 121 ESBL-producing *K. pneumoniae* isolates originally recovered during 2005–2009, *bla*_SHV_ and *bla*_TEM_ genes were detected in 102 (84.3%) and 61 (50.4%) of 121 isolates_,_ respectively (Table 1). Overall, 25 CTX-M-type ESBL *K. pneumoniae* were identified. However, none of the 81 *K. pneumoniae* isolates from 2005 through 2008 was positive for *bla*_CTX-M_ genes. CTX-M–type ESBL was first detected in 2 (5.0%) of 40 *K. pneumoniae* isolates from 2009. The prevalence of *K. pneumoniae* isolates carrying the CTX-M–encoding genes increased to 6 (17.6%) of 34 in 2010 and 12 (34.3%) of 35 in 2011. The level remained high (27.8%, 5/18) in the first 7 months of 2012. Overall, only 2 (1.7%) of 121 ESBL-producing *K. pneumoniae* isolates from 2005 through 2009 carried the *bla*_CTX-M_ genes, compared with 23 (26.4%) of 87 isolates from 2010 through 2012 (p<0.0001, Fisher exact test), indicating the rapid emergence and spread of CTX-M enzymes among ESBL-producing *K. pneumoniae* clinical isolates since 2009.

**Table 1:** Detection of *bla*_ESBL_ genes of the SHV, TEM, and CTX-M types in 208 ESBL-producing *Klebsiella pneumoniae* clinical isolates, 2005–2012*

Year	No. isolates tested	No. (%) positive isolates			CTX-M type (no. isolates)
*bla*_SHV_	*bla*_TEM_	*bla*_CTX-M_
2005	22	20 (90.9)	7 (31.8)	0	
2006	21	15 (71.4)	11 (52.4)	0	
2007	17	11 (64.7)	10 (58.8)	0	
2008	21	19 (90.5)	10 (47.6)	0	
2009	40	37 (92.5)	23 (57.5)	2 (5.0)	CTX-M-15 (2)
2010	34	31 (91.2)	9 (26.4)	6 (17.6)	CTX-M-15 (4), CTX-M-2 (1), CTX-M-3 (1)
2011	35	32 (91.4)	13 (36.1)	12 (34.3)	CTX-M-15 (9), CTX-M-3 (2), CTX-M-1 (1)
2012	18	16 (88.9)	8 (44.4)	5 (27.8)	CTX-M-15 (4), CMX-M-3 (1)
Total	208	181 (87.0)	91 (43.8)	25 (12.0)	
* ESBL, extended-spectrum β-lactamase					

### CTX-M in ESBL-producing *E. coli* Clinical Isolates

One hundred sixty-three ESBL-producing *E. coli* clinical isolates from 2005 through 2012 were analyzed by PCR for detection of *bla*_ESBL_ genes of the SHV, TEM, and CTX-M types (Table 2). Unlike the situation with *K. pneumoniae*, *bla*_CTX-M_ genes were detected in ESBL-producing *E. coli* isolated as early as 2005. Overall, 89 (54.6%) of 163 ESBL *E. coli* isolates from the 8-year period carried *bla*_CTX-M_ genes. CTX-M was the leading ESBL type in all years examined except 2008. The *bla*_CTX-M_ genes from 47 (52.8%) of 89 CTX-M–producing *E. coli* isolates were sequenced. CTX-M-15 was determined in 45 (95.7%) of 47 CTX-M–producing *E. coli* isolates analyzed. CTX-M-1 and CTX-M-3 genotypes were each found in 1 ESBL *E. coli* isolate.

**Table 2:** Detection of *bla*_ESBL_ genes of the SHV, TEM, and CTX-M types in 163 ESBL-producing *Escherichia coli* clinical isolates, 2005–2012

Year	No. isolates tested	No. (%) positive isolates			CTX-M type (no. isolates/total no. isolates sequenced)
		*bla*_SHV_	*bla*_TEM_	*bla*_CTX-M_	
2005	20	6 (30.0)	4 (20.0)	7 (35.0)	CTX-M-15 (5/5)
2006	16	1 (6.3)	3 (18.8)	16 (56.3)	CTX-M-15 (6/6)
2007	24	4 (16.7)	9 (37.5)	10 (50.0)	CTX-M-15 (4/6), CTX-M-1 (1/6), CTX-M-3 (1/6)
2008	20	5 (25.0)	10 (50.0)	6 (30.0)	CTX-M-15 (5/5)
2009	22	0 (0)	12 (54.5)	13 (59.1)	CTX-M-15 (5/5)
2010	20	1 (5.0)	9 (45.0)	13 (65.0)	CTX-M-15 (6/6)
2011	21	3 (14.3)	9 (42.9)	11 (52.3)	CTX-M-15 (8/8)
2012	20	2 (10.0)	10 (50.0)	13 (65.0)	CTX-M-15 (6/6)
Total	163	22 (13.5)	66 (40.5)	89 (54.6)	

*ESBL, extended spectrum β-lactamase

### CTX-M in KPC-producing *K. pneumoniae* Clinical Isolates

Two hundred twenty-eight KPC-producing *K. pneumoniae* isolates from 2005 to 2012 were examined by PCR for detection of *bla*_CTX-M_ genes. All *K. pneumoniae* isolates were positive for the *bla*_KPC_ gene by PCR as described (*22*). None was positive for the *bla*_CTX-M_ gene.

### Clinical and Microbiological Characteristics of CTX-M–producing *K. pneumoniae*

Selected clinical/epidemiologic features of the 25 patients with CTX-M ESBL–producing *K. pneumonia* and certain microbiological characteristics of the isolates are shown in Table 3, Appendix. Mean patient age was 56 years, and 13 (52%) of the patients were male. Sixteen patients (64%) had bacteriuria. CTX-M–producing *K. pneumoniae* isolates were recovered from 13 (52%) patients within 72 hours of hospital admission; however, 18 (72%) of these patients had been hospitalized in the 8 months before the current admission.

**Table 3:** Selected clinical/epidemiologic features of the 25 patients with CTX-M ESBL–producing *K. pneumoniae* from New York, USA and certain microbiological characteristics of the isolates*

Year	Isolate	Patient age, y	Sex	Source	≤72 h of admission	Prior hospital admission^†^	Prior ESBL *KP/E. coli*^†^	LOS, d‡	Disk diffusion (mm)		MIC (μg/mL)		β-lactamase(s)	PFGE type (n = 17)	ST type (n = 18)
									CTX	CAZ	CTX	CAZ			
2009	T76	93	M	Urine	N	Y	N	11	9	18	>256	8	CTX-M-15, SHV-28, TEM-1	PF5	15
	PK114	72	F	Urine	Y	Y	N	5	ND	ND	ND	ND	CTX-M-15, SHV-11, TEM-1	ND	437
2010	KF351	24	M	Urine	N	Y	N	7	8	21	>256	3	CTX-M-2, SHV-11, TEM-1	PF6	792
	KF403	71	F	Urine	Y	Y	Y/Y	10	6	13	>256	32	CTX-M-15, SHV-11, TEM-1	PF6	437
	KF548	31	M	Blood	N	Y	N	20	10	15	>256	16	CTX-M-15, SHV-27, TEM-1	PF1	280
	KF563	57	M	Respiratory	N	Y	Y	20	6	6	>256	96	CTX-M-15, SHV-28, TEM-1	PF2	15
	KF602	70	M	Urine	N	N	N	3	8	14	128	12	CTX-M-15, SHV-11, TEM-1	PF7	17
	PK6	71	M	Respiratory	N	Y	N/Y	4	ND	ND	ND	ND	CTX-M-3, SHV-11, TEM-1	ND	ND
2011	KG318	64	F	Urine	Y	Y	Y	8	6	12	>256	64	CTX-M-15	PF8	ND
	KG291	67	M	Urine	N	N	N	19	ND	ND	ND	ND	CTX-M-15, SHV-11, TEM-1	ND	ND
	KG304	44	F	Urine	Y	Y	Y	21	6	9	>256	32	CTX-M-15, SHV-1, TEM-1	PF8	252
	KP33	71	F	Urine	N	Y	N	13	10	17	>256	8	CTX-M-15, SHV-1, TEM-1	PF3	16
	KP38	69	M	Urine	Y	Y	N	7	10	19	>256	6	CTX-M-1, SHV-11	PF3	11
	KP34	62	M	Urine	Y	Y	Y	12	13	22	32	3	CTX-M-15, SHV-11	PF5	147
	KP41	7	F	Wound	Y	Y	N	1	6	18	>256	12	CTX-M-15, SHV-1, TEM-1	PF4	ND
	KP35	39	F	Urine	Y	N	N	1	6	13	>256	32	CTX-M-15, SHV-11	PF5	392
	KP37	44	M	Blood	Y	Y	N	5	6	10	>256	48	CTX-M-15, SHV-1	PF4	15
	PK23	15	M	Respiratory	Y	N	N	5	10	18	128	6	CTX-M-15, SHV-11, TEM-1	PF4	48
	PK30	62	F	Respiratory	N	N	N	26§	9	8	>256	16	CTX-M-3, SHV-11, TEM-1	PF3	ND
	PK107	71	F	Blood	N	N	N	90§	8	8	>256	>256	CTX-M-3, SHV-11, TEM-1	PF3	11
2012	PK133	52	M	Urine	Y	Y	N	90	6	15	128	24	CTX-M-15, SHV-11	ND	ND
	PK135	81	F	Urine	N	N	N	14	11	20	64	6	CTX-M-3, SHV-11, TEM-1	ND	11
	PK140	66	F	Urine	Y	Y	N/Y	7	7	15	>256	16	CTX-M-15, SHV-11	ND	392
	CK17	34	F	Urine	Y	N	N	26	16	8	16	>256	CTX-M-15, SHV-12	ND	258
	CK30	53	M	Wound	N	N	N	77	6	15	>256	48	CTX-M-15, SHV-1, TEM-1	ND	nd

*ESBL, extended-spectrum β-lactamase; ; CTX: cefotaxime; CAZ: ceftazidime; PFGE, pulsed-field gel electrophoresis; ST, sequence type; Y, yes; N, noND, not determined. †Prior hospitalization or history of recovering ESBL *K. pneumoniae* (KP) and *E. coli* isolate within 8 mo of the current admission. ‡LOS, length of stay after the recovery of CTX-M *K. pneumoniae*.  §Patients died.

The *bla*_CTX-M_ genes from all 25 CTX-M ESBL–producing *K. pneumoniae* isolates from 2009 through 2012 were sequenced. CTX-M-15 was identified in 19 (76.0%) and was the dominant CTX-M genotype. The remaining 6 isolates were determined to be CTX-M-3 (n = 4), CTX-M-1 (n = 1), and CTX-M-2 (n = 1), respectively. Twenty-four (96.0%) had coexisting *bla*_SHV_ β-lactamases, which were predominantly non-ESBL *bla*_SHV-11_ (n = 15) and *bla*_SHV-1_ (n = 5)_._ Four additional *K. pneumoniae* carried ESBL-type *bla*_SHV_ β-lactamases, including *bla*_SHV-12_ (n = 1), *bla*_SHV-27_ (n = 1), and *bla*_SHV-28_ (n = 2). Seventeen (68.0%) were positive for TEM-type β-lactamases, and all were confirmed to be *bla*_TEM-1._

The antimicrobial drug susceptibilities of CTX-M–producing *K. pneumoniae* isolates are summarized in Table 4. Of the 25 CTX-M–producing *K. pneumoniae* isolates examined in this study, only 12% (n = 3) and 36% (n = 8) of isolates were susceptible to ciprofloxacin and gentamicin, respectively. Low susceptibility rates were also observed for pipercillin/tazobactam (36%), tetracycline (20%) and trimethoprim/sulfamethoxazole (4%). Twenty-three of the 25 (92%) isolates tested were susceptible to carbapenems. Notably, the 2 carbapenem-resistant *K. pneumoniae* isolates (PK30 and PK107) carried *bla*_CTX-M-3,_ *bla*_SHV-11,_ and *bla*_TEM-1._ One of these *K. pneumoniae* isolates also showed resistance to colistin with an MIC of 64µg/mL. Both patients died of complications associated with bloodstream and respiratory tract infections. Three of 22 CTX-M–producing *K. pneumoniae* isolates examined by Etest were nonsusceptible to tigecycline (MICs 3 µg/mL, 3 µg/mL, and 8 µg/mL).

**Table 4:** In vitro antimicrobial susceptibility of CTX-M ESBL-producing *K. pneumoniae* isolates, New York, 2005–2012*

Antimicrobial agent	No. isolates tested	No. (%) susceptible isolates	MIC_50_	MIC_90_	MIC range
Cefoxitin	25	16 (64.0)	≤8	>16	<8–>16
Cefotaxime†	22	0	>256	>256	16–>256
Ceftazidime†	22	2 (9.1)	16	128	4–>256
Pip/Tazo	25	9 (36.0)	64	>64	<16–>64
Ertapenem	25	23 (92.0)	<2	<2	<2–>4
Meropenem†	22	21 (95.5)	0.094	0.125	0.047–2.0
Imipenem†	22	20 (90.1)	0.25	1.5	0.19–6.0
Ciprofloxacin	25	3 (12.0)	>2	>2	<1–>2
Amikacin	25	18 (72.0)	<16	>32	<16–>32
Gentamicin	25	8 (32.0)	>8	>8	<4–>8
Tetracycline	25	5 (20.0)	>8	>8	<4–>8
TMP/SMX	25	1 (4.0)	>2/38	>/38	<2/38–>2/38
Tigecycline†‡	22	19 (86.4)	1	3	0.75– 8
Colistin†§	22	21 (95.5)	0.25	0.38	0.19–64

*n = 25; MIC_50_; 50% minimum inhibitory concentration; MIC_90_, 90% minimum inhibitory concentration; Pip/Tazo, piperacillin/tazobactam; TMP/SMX, trimethoprim/sulfamethoxazole. MICs were determined by the MicroScan system, except for certain antimicrobial agents that were tested by Etest as specified. †MICs were determined by Etest. ‡Susceptibility defined by Food and Drug Administration breakpoints. §Susceptibility defined by Clinical Laboratory and Standards Institute breakpoints for *Acinetobacter baumannii* (*19*).

**Figure 1:**
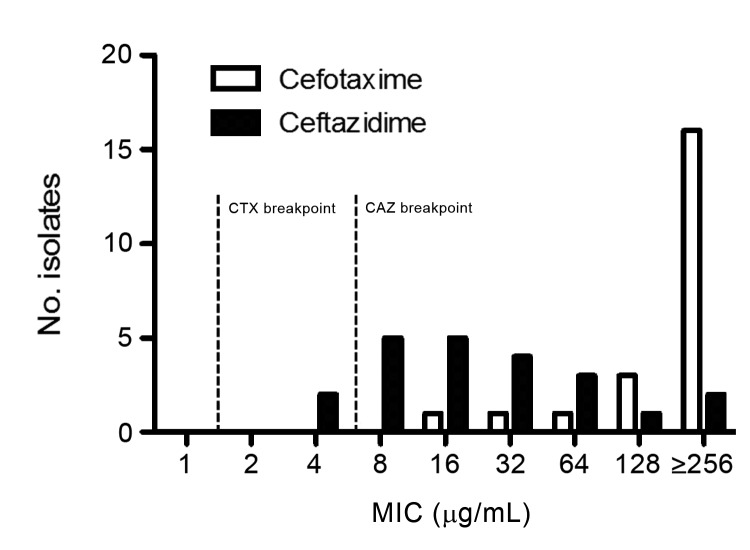
MIC distribution for cefotaxime (CTX) and ceftazidime (CAZ) in CTX-M extended-spectrum β-lactamase–producing *Klebsiella pneumoniae* clinical isolates from a tertiary care medical center in suburban New York, NY, USA, 2005–2012 (n = 22). The MICs were determined by Etest.

All 25 CTX-M–producing *K. pneumoniae* isolates examined were resistant to cefotaxime, and all but 1 isolate showed higher MICs of cefotaxime than of ceftazidime. The 50% minimum inhibitory concentration (MIC_50_) for cefotaxime among these isolates was >256 µg/mL. By contrast, the MIC_50_ and 90% inhibitory concentration for ceftazidime were 16 µg/mL and 128 µg/mL, respectively. Two CTX-M–producing *K. pneumoniae* isolates (8.0%) were susceptible (MIC ≤4 µg/mL) and 5 isolates (20%) were intermediate in susceptibility (8 µg/mL) to ceftazidime according to the 2010 revised CLSI breakpoints (Figure 1). In addition, we determined the susceptibilities of 22 CTX-M–producing *K. pneumoniae* isolates against cefotaxime and ceftazidime by using the standard disk diffusion method. All CTX-M–producing *K. pneumoniae* isolates examined were resistant to cefotaxime by disk diffusion (mean inhibitory zone size 8.3 mm; range 6–13 mm). Two of these isolates were susceptible (≥21 mm) and 5 had intermediate (18–20 mm) susceptibility to ceftazidime by disk diffusion (Table 3, appendix). The disk diffusion results showed a category agreement with the Etest MIC of 100% for cefotaxime and 90.9% for ceftazidime with 2 minor errors.

### PFGE and MLST Analysis of CTX-M–producing *K. pneumoniae*

**Figure 2:**
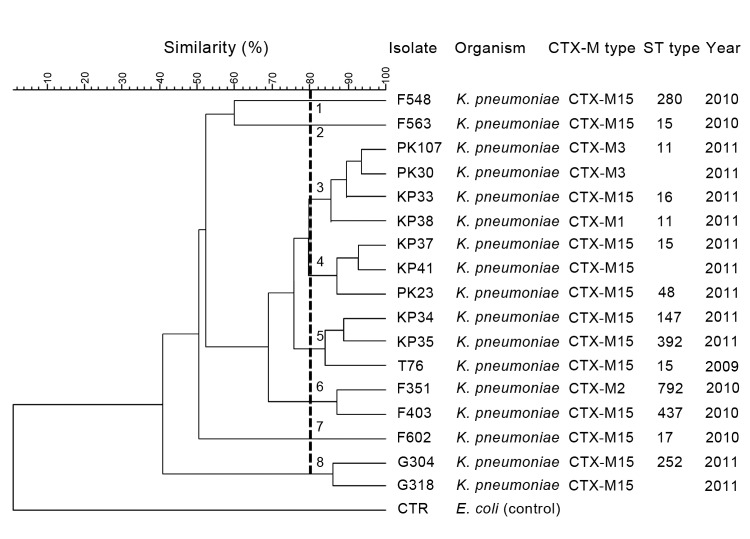
Dendrogram of pulsed-field gel electrophoresis (PFGE) patterns showing the genetic relatedness of CTX-M extended-spectrum β-lactamase (ESBL)–producing *Klebsiella pneumoniae* isolates from patients in suburban New York, NY, USA (n = 17). Eight PFGE pulsetypes (PF1–8) were identified with ≥80% similarity, which is marked by the vertical line. The corresponding CTX-M genotype, sequence type (ST), if available, and year of isolation for each isolate are listed on the right side of the dendrogram.

Of 17 representative CTX-M-producing *K. pneumoniae* isolates analyzed by PFGE, 8 different pulsotypes (PF1–8) were identified with Dice coefficients of ≥80% similarity (Figure 2). Ten of 17 *K. pneumoniae* isolates examined belonged to 3 major groups (PF3, PF4, PF5) with 3–4 isolates in each group. The remaining pulsotypes contained only 1 or 2 *K. pneumoniae* isolates. No clear temporal relationship was shown among the highly related isolates.

MLST was performed on 18 CTX-M–producing *K. pneumoniae* isolates. These isolates were selected to represent different CTX-M genotypes, pulsotypes, antimicrobial susceptibility profiles, and years of isolation. Twelve STs were recognized for the *K. pneumoniae* isolates examined (Table 3, appendix). Notably, all 3 CTX-M group 1, non–CTX-M-15 *K. pneumoniae* isolates analyzed (KP38, PK107, and PK135) had ST11, whereas 10 different STs (ST15, ST16, ST17, ST48, ST147, ST252, ST258, ST280, ST392, and ST437) were identified for the 14 CTX-M-15 *K. pneumoniae* isolates. Isolate F351 was the only non–CTX-M-1 group *K. pneumoniae* isolate identified in this study and was determined to be a separate group (ST792) by MLST. Of the 14 CTX-M–producing *K. pneumoniae* isolates evaluated simultaneously by DNA sequencing, PFGE and MLST, a high genetic divergence was demonstrated by the detection of 4 CTX-M genotypes (CTX-M-1, CTX-M-2, CTX-M-3, and CTX-M-15), 8 pulsotypes (PF1–8) and 11 STs (ST11, ST15, ST16, ST17, ST48, ST147, ST252, ST280, ST392, ST437, and ST792) (Figure 2).

## Discussion

CTX-M ESBL–producing *E. coli*, especially ST131 strains, have emerged in recent years in several US states (*5*–*7*,*25*,*26*). In this study, we detected *bla*_CTX-M_ genes in ESBL-producing *E. coli* strains isolated from patients at a tertiary care medical center in suburban New York City as early as 2005. Eighty-nine (54.6%) of 163 ESBL-producing *E. coli* isolates in the study period (2005–2012) carried *bla*_CTX-M_. Our findings confirm the emergence and dominance of CTX-M enzymes in ESBL-producing *E. coli* since the mid-2000s in the New York City metropolitan area (*9**,**10*). 

Despite this high prevalence of CTX-M in ESBL-producing *E. coli* since 2005, none of 81 ESBL-producing *K. pneumoniae* isolates recovered from patients at the same tertiary care medical center from 2005 through 2008 was positive for *bla*_CTX-M_. CTX-M-type ESBL was first detected in *K. pneumoniae* isolates from our institution in 2009. The percentage of *K. pneumoniae* isolates carrying *bla*_CTX-M_ has increased significantly since then. During 2010–2012, *bla*_CTX-M_ genes were identified in 23 of 87 (26.4%) ESBL-producing *K. pneumoniae* isolates. These data demonstrate the rapid emergence and spread of CTX-M ESBL-producing *K. pneumoniae* in our patients. To date, CTX-M–producing *K. pneumoniae* has been recognized in several US states, including Texas (2004–2007, n = 11) (*5*,*12*), Nebraska (2005, n = 1) (*15*), Pennsylvania (2007, n = 5) (*11*), and 1 isolate in 2007 each from California, Massachusetts, Michigan, New Jersey, New York, Washington, and Wisconsin (*12*). In addition, a few CTX-M *K. pneumoniae* isolates have been reported from 2 collections of the SMART surveillance program with isolates recovered during 2008–2009 (*16*) and 2009–2010 (*18*). No CTX-M was detected in US ESBL-producing *K. pneumoniae* isolates collected before 2000 (*3*), with all CTX-M–producing *K. pneumoniae* isolates recovered from US patients in or after 2004. Therefore, we speculate that the emergence and spread of *bla*_CTX-M_ in *K. pneumoniae* are recent evolutionary events that most likely occurred in the mid- to late-2000s in the United States.

The particular CTX-M enzyme type in ESBL-producing *K. pneumoniae* and *E. coli* varies geographically. CTX-M-15, which belongs to the CTX-M-1 group, is the most prevalent CTX-M allele with a worldwide distribution (*1**,**2**,**26*). CTX-M-14, which belongs to the CTX-M-9 group, is another common variant that is highly prevalent in some European and Asian countries (*27**–**30*), whereas CTX-M-2 in the CTX-M-2 group and CTX-M-8 seem to be dominant in South America (*1**,**31*). In the United States, CTX-M-15 is the most frequently detected genotype among CTX-M–producing *K. pneumoniae* isolates, followed by CTX-M-14 (*5**,**11**,**12*). CTX-M-2 group and CTX-M-8 group ESBL-producing *K. pneumoniae* each was identified in 1 isolate (*16*).

Our data provide strong evidence for the recent, rapid emergence, and polyclonal spread of the CTX-M-1 group, especially CTX-M-15 ESBL-producing *K. pneumoniae* in a US hospital setting. In this study, 24 (96.0%) of 25 *bla*_CTX-M_-positive *K. pneumoniae* were categorized as group 1 CTX-M, including isolates encoding CTX-M-15 (n = 19), CTX-M-1 (n = 1), and CTX-M-3 (n = 4). Similarly, group 1 CTX-M was detected in 47 (100%) of 47 *bla*_CTX-M_-positive *E. coli* isolates. In addition, 1 *K. pneumonia* isolate had the CTX-M-2 genotype. No CTX-M-14 was detected in these *K. pneumoniae* and *E. coli* isolates. CTX-M-14 has been reported in *E. coli* ESBL isolates in several US states, including geographically adjacent Pennsylvania (*6*). CTX-M-14 has also been reported in *K. pneumoniae* isolates in the Calgary Healthcare Region of Canada (*32*). Why CTX-M-14 is absent in the ESBL-producing *E. coli* and *K. pneumoniae* isolates from the New York, NY metropolitan area is unknown. Because CTX-M-15–producing *K. pneumoniae* isolates may exhibit significantly higher resistance rates to ciprofloxacin and pipercillin-tazobactam than CTX-M-14–producing isolates (*27**,**28*), CTX-M genotypes and their antimicrobial drug profiles should be monitored among CTX-M–producing *E. coli* and *K. pneumoniae* isolates in regions where they are emerging.

We investigated the genetic relatedness of CTX-M–producing *K. pneumoniae* isolates by PFGE and MLST. Of the 17 representative isolates examined by PFGE, 8 different pulsotypes were determined. Similarly, 12 MLST STs were identified for the 18 CTX-M–producing isolates analyzed. Our data, in combination with findings from other groups (*1*), suggest that CTX-M–producing *K. pneumoniae* isolates are genetically heterogeneous. The emergence and polyclonal spread of CTX-M–producing *K. pneumoniae* likely occurred among isolates with diverse genetic backgrounds. This hypothesis contrasts with findings regarding KPC-producing *K. pneumoniae*: a clonal spread of KPC-producing *K. pneumoniae* isolates belonging to the ST258 lineage was observed by us (*33*) and Pournaras et al. (*34*). In clinical strains, CTX-M–encoding genes have commonly been located on plasmids that vary in size from 7 kb to 160 kb (*2*). Plasmid-mediated transmission of CTX-M genes in *Enterobacteriaceae* that involves several motile genetic elements has been described (*2**,**35**,**36*). Given the dominance of CTX-M-15 genotypes among genetically heterogeneous *K. pneumoniae* isolates, our study also implies the probable horizontal transfer of a genetic element carrying *bla*_CTX-M_ among *K. pneumoniae* isolates.

Of the 12 STs determined for the CTX-M ESBL–producing *K. pneumoniae* isolates, ST11, ST15, ST17, ST48, ST147, and ST258 have been reported in CTX-M–positive *K. pneumoniae* in Spain, Hungary, or Korea (*28**,**37**,**38*). Among these, only ST17 was reported among CTX-M–producing *K. pneumoniae* isolates in Canada (*39*). In this study, we determined the STs among CTX-M–producing *K. pneumoniae* in the United States and document the existence of 6 STs (ST16, ST252, ST280, ST392, ST437, ST792) in CTX-M–producing *K. pneumoniae* not previously described.

The CTX-M–producing *K. pneumoniae* isolates evaluated in this study showed several notable epidemiologic, clinical, and microbiological features. First, most CTX-M–producing isolates were recovered from patients with bacteriuria, which is similar to that observed for infections caused by CTX-M–producing *E. coli* in New York City (*9**,**10*). Although CTX-M–producing *K. pneumoniae* was isolated in clinical specimens collected within 72 hours of hospitalization in about half of the patients, 18 (72%) of 25 patients had been hospitalized in the prior 8 months. This factor highlights the potential for acquiring CTX-M–producing *K. pneumoniae* in health care settings and differs from the experience with CTX-M–producing *E. coli* that are associated with infections arising in the community setting unrelated to exposure to health care facilities (*26*). Second, the CTX-M–producing *K. pneumoniae* study isolates exhibited high rates of resistance to gentamicin (68%), trimethoprim-sulfamethoxazole (96%), and tetracycline (80%), in addition to resistance to ciprofloxacin (88%) and pipercillin-tazobactam (64%) as described previously in Europe and Asia (*27**,**28**,**37*). Whether such high rates of resistance are associated with the dominant spread of CTX-M-15–, rather than CTX-M-14–producing *K. pneumoniae,* in these patients is not known. The coexistence of CTX-M ESBL and TEM-1 and SHV-type β-lactamases in these isolates may have also contributed to the observed high rate of antimicrobial drug resistance. All except 1 of our CTX-M–positive *K. pneumoniae* isolates produced SHV- and CTX-M–type ESBLs. These findings have clinical implications for selecting empiric antimicrobial drug therapy when infection caused by ESBL-producing *K. pneumoniae* is suspected. The rapid emergence of such CTX-M–producing *K. pneumoniae* isolates, mainly in US hospitals, is also raising new concerns for public health and infection control practice. Third, none of the 228 KPC-producing *K. pneumoniae* isolates examined carried *bla*_CTX-M._ Coexistence of *bla*_KPC_ and *bla*_CTX-M_ has only been reported in KPC-producing *K. pneumoniae* in China (*40*). Whether certain genetic mechanisms prevent KPC-producing *K. pneumoniae* from acquiring *bla*_CTX-M_ is unclear.

This study reveals the rapid emergence and polyclonal spread of CTX-M–producing *K. pneumoniae* in patients in Westchester County, New York. A limitation of our study is that the clinical isolates were collected from patients at a single tertiary-care medical center. Investigations of CTX-M–producing *K. pneumoniae* isolates from a variety of geographic regions should be undertaken to clarify the epidemiology and clinical and public health effects of the emergence of CTX-M–producing *K. pneumoniae* in the United States.
